# Study of the Structure and Infrared Spectra of LiF, LiCl and LiBr Using Density Functional Theory (DFT)

**DOI:** 10.3390/ma16155353

**Published:** 2023-07-30

**Authors:** Katarzyna Chruszcz-Lipska, Elżbieta Szostak, Krzysztof Kazimierz Zborowski, Ewa Knapik

**Affiliations:** 1 Faculty of Drilling, Oil and Gas, AGH University of Science and Technology, Mickiewicza 30 Ave., 30-059 Kraków, Poland; eknapik@agh.edu.pl; 2 Faculty of Chemistry, Jagiellonian University in Kraków, Gronostajowa 2 Str., 30-387 Kraków, Polandzborowsk@chemia.uj.edu.pl (K.K.Z.)

**Keywords:** far infrared spectroscopy, DFT calculation, lithium halides: LiF, LiCl, LiBr, ATP charge distribution

## Abstract

The paper presents a study of the crystal structure of anhydrous halides LiF, LiCl and LiBr using density functional theory. Models composed of 125 atoms were used for this study. The theoretical values of the lattice parameters and the distribution of charges in the crystals were determined. Using the assumed models at the level of theory DFT/B3LYP/6-31+g*, the theoretical infrared spectra of lithium halides (LiF, LiCl and LiBr) were calculated for the first time. Additionally, measurements of experimental far-infrared (FIR) spectra were performed for these salts. All the obtained theoretical values were compared with experimental data obtained by us and those available in the literature.

## 1. Introduction

The industrial importance of lithium compounds is growing due to the development of electromobility and advanced energy storage systems [1,2,3]. More than 70% of all produced lithium is used in the manufacturing of Li-ion batteries for electronics and electric vehicles [4]. Other global end-use markets are estimated as follows: ceramics and glasses, 14%; greases, 3%; continuous casting mold flux powders, 2%; polymer production, 2%; air treatment, 1%; and others, 4%. Lithium is traded mainly in the form of two components, Li_2_CO_3,_ which accounts for 46% of the total quantity (in 2015), and LiOH (19%) [5]. High-purity lithium carbonate is well described in the literature (in terms of its preparation [6,7] and characterization using spectral methods [8,9]). The IR spectra for LiOH have been known for more than 50 years [10], and some new aspects, like LiOH hydration behavior, are still being studied [11,12]. Of the various lithium compounds, lithium halides (LiF, LiCl and LiBr) are rarely studied, despite their various industrial applications. 

LiCl is the most common lithium compound that occurs in continental brines, geothermal waters and oilfield brines [13]. For practical reasons, LiCl is converted to Li_2_CO_3_ (Li_2_CO_3_ is precipitated from LiCl-saturated solutions) [14,15]. LiCl is highly hygroscopic and undergoes the phenomenon of deliquescence, which means that it sorbs water vapor from the air and spontaneously becomes an aqueous solution. This unique feature allows it to be used as a desiccant for air-conditioning and drying purposes [16], or it can even be used to produce potable water by capturing water vapor from atmospheric air [17]. At the same time, this hygroscopicity hinders the application of LiCl in low-temperature heat storage systems [18]. To overcome this drawback, various vapor-permeable composite materials have been developed [19,20]. Other applications of lithium chloride include fluxes for brazing aluminum [21], catalysts for organic oxidation reactions [22], separation of RNA in biological systems [23], medical treatments for bipolar disorder [24], and molten salt mixtures [25,26]. Lithium bromide solutions are used mainly in air cooling systems’ absorption chillers, where water is the refrigerant and a solution of LiBr acts as the absorbent [27,28]. Chillers of this kind can provide useful room cooling using waste heat or renewable energy [29,30]. Highly concentrated (50–60%) LiBr brines are employed as liquid desiccants [31]. Solid LiBr is often used in organic synthesis [32,33] and the catalytic conversion of waste biomass [34,35,36]. Unlike heavier halides, LiF dissolves poorly in water [37] and has rather niche applications, such as radiation-shielding materials [38], glasses for photonic applications [39] and optical materials [40].

Lithium halides are thus widely used in different classes of materials, including glass, polymers (plastics), composites, catalysts and alloys. Regardless of their form (anhydrous solids or aqueous solutions), vibrational spectroscopy may be employed to assess their purity. Our previous study [41] showed that far-infrared spectroscopy is a useful tool to distinguish NaCl and KCl minerals, and even blue and colorless NaCl from Kłodawa. Although the structural, elastic, electronic, etc., properties of LiF, LiCl, LiBr, and LiI compounds have been studied, we have noticed that the far-infrared spectra of anhydrous lithium halides have not yet been reported in the literature. There are numerous studies on halide solvation using both experimental and computational infrared spectroscopy [42,43,44]; however, the baseline, i.e., FIR spectra, for anhydrous salts has not yet been shown. DFT calculations are often performed to evaluate the structural and electrochemical properties of various cathode materials for lithium batteries. The first ab initio calculations for a cathode material Li_2_CoMn_3_O_8_ (high-energy and voltage, rechargeable 5 Volt Li-ion battery) were performed by Eglitis and Borstel [45]. Our aim is to fulfill the gap and present the calculated IR spectra of the crystal structure models of lithium halides obtained using the density functional theory (DFT). In addition, our experimental work verifies the calculated spectra. Our results may contribute to the advancement of material characterization using spectral techniques and prove the applicability of FIR in the differentiation of lithium halides.

## 2. Materials and Methods

### 2.1. Chemical Reagents

Lithium fluoride (LiF), chloride (LiCl) and bromide (LiBr) (analytical grade, 99+%, ACS reagent) anhydrous salts were purchased from Thermo Fisher Scientific and ROTH. Immediately after opening the original manufacturer’s packing and without any preparation, the chemical reagents were taken for the infrared spectra measurements. 

### 2.2. DFT Calculations

Calculations were performed at the DFT level, using the B3LYP functional and 6–31+g* basis set, using the Gaussian’16 program packages [46,47,48]. B3LYP is one of the most commonly used functionals. Eglitis et al.’s [49] calculations show that the B3PW or B3LYP hybrid exchange-correlation functionals make it possible to achieve excellent agreement with experiment for the band gaps for BaF_2_, SrF_2_ and CaF_2_. The input geometries of salt crystal models (LiF, LiCl and LiBr) were taken for calculation from crystallographic data [50,51,52]. Our previous study showed that calculations for a 125-atom model for NaCl and KCl give reasonable results [41]. This 125-atom structure provides that each of the 27 atoms of the conventional unit cell of the studied lithium halides has six-fold coordination. Moreover, according to our previous study for NaCl and KCl, freezing of all bond angles to 90° in the structure during the geometry optimization process leads to a good simulation of the lattice parameters of the crystals and their infrared spectra [41]. Thus in this work, the geometries of the 125-atom model structures of LiF, LiCl and LiBr were fully optimized with the restriction for all angles 90°. No imaginary frequencies were determined for any of the optimized structures of the tested halides of alkali metals. Standard conditions (temperature of 298.15 K and pressure of 1 atm.) were applied. The IR spectra of LiF, LiCl and LiBr were calculated by representing each band as a Lorentzian-shaped curve. Half-bandwidths of 50 cm^−1^ were used to take temperature broadening into account. Calculated frequencies were scaled by 0.9 factor [53]. The atomic charge is not a quantum chemical observable; therefore, there are many methods for dividing the total charge of a molecule into atomic charges, of which one of the most well-known and widely used is the APT (Atomic Polar Tensors) method [54]. In this approximation, the atomic charge is related to the trace of the corresponding atomic polar tensor, i.e., the tensor of the derivatives of the dipole moment with respect to atomic Cartesian coordinates.

### 2.3. Far Infrared Spectroscopy

The infrared absorption spectra measurements were taken on a Bruker VERTEX 70v FT-IR spectrometer. Spectra were recorded in room conditions in the spectral range of 620–30 cm^−1^. The samples of lithium halides (LiF, LiCl, LiBr) were suspended in Apiezon grease and placed on a polyethylene window. The spectra were measured in triplicate with 32 scans and a resolution of 2 cm^−1^.

## 3. Results

### 3.1. Model of Crystals of Anhydrous Salts: LiF, LiCl and LiBr

#### 3.1.1. Geometry

**Figure 1:**
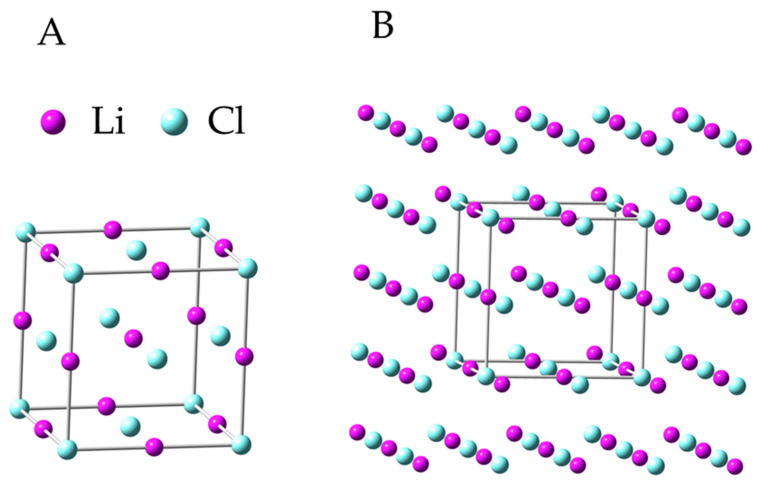
Unit cell of LiCl (**A**) and geometry of optimized 125-atom model of LiCl crystal (DFT/B3LYP/6-31+g*, all angles equals 90°) (**B**).

The first step of the quantum-chemical calculation was geometry optimization of the model of crystals of anhydrous salts LiF, LiCl and LiBr. The lithium fluoride, chloride and bromide crystallize in $Fm\bar{3}m$ space-group symmetry. In the Schoenflies system, it is O*h* point group. The initial 125-atom model of the LiF (LiCl and LiBr) crystal that was taken for calculations was part of the crystal structure determined by X-ray measurements [50,51,52]. Figure 1A shows the LiCl unit cell (same for LiCl and LiBr), which contains 27 atoms (3 × 3 × 3). In order to preserve the symmetry of the unit cell and at the same time guarantee that each of the 27 atoms of this unit cell had 6-fold coordination, a model containing 125 atoms (5 × 5 × 5) had to be created (Figure 1B). This model was built by adding one more layer of atoms on each of the six faces of a unit cell. During geometry optimization, there was no change in the symmetry of the assumed models, and the final structures had O*h* symmetry.

A comparison of the experimental and calculated geometrical parameters for LiF, LiCl and LiBr unit cells is presented in Table 1.

The results shown in Table 1 indicate that the theoretical values (calculation for 264 distances of Li-halide and for six distances of Li-halide in the surrounding of the central 6-coordinated Li atom) are lower than those determined by X-ray diffraction. The theoretical values are slightly smaller than the experimental values because they correspond to a static crystal. The lattice parameters of lithium halides increase in the order LiF < LiCl < LiBr, which corresponds with an increase in the atomic radius of the halogen. The value of the cell parameter equals a = 5.038 Å and a = 5.283 Å for LiCl and LiBr, respectively. These values are very similar, which is certainly due to the similar value of the electronegativity (E) and ionic radius (R) [55] for these elements (E_Cl_ = 2.83, R_Cl−_ = 181 pm; E_Br_ = 2.74, R_Br−_ = 196 pm). The value of this cell parameter for LiF is significantly different: 3.906 Å (E_F_ = 4.1, R_Cl−_ = 119 pm). The results obtained during the calculations are fully consistent with the experimental data.

#### 3.1.2. Charge Distribution in Model Structure of LiF, LiCl and LiBr 

Table 2 shows the calculated ATP charge (DFT/B3LYP/6–31+g*) on the lithium atom and halide atoms in each model structure of LiF, LiCl and LiBr. The value of the negative charge on the atom of the appropriate halide decreases in accordance with the series F > Cl > Br and equals −0.8198, −0.7796 and −0.7408, respectively. This is consistent with the decreasing value of electronegativity of individual elements. The positive charge on the lithium atom is +0.8198, +0.7796 and +0.7408 for LiF, LiCl and LiBr, respectively. The energetic conditions near/on the surface of the crystal are different from those inside it. Inside the solid, the forces acting on the ions are balanced. On the surface, not all coordination sites are saturated, which creates slightly different conditions and determines reactivity. As shown in Table 2, there is a noticeable difference between the charge on individual ions inside the crystal model and on its surface.

### 3.2. Infrared Spectra of LiF, LiCl and LiBr

For the previously optimized structures (models of LiF, LiCl and LiBr), the IR frequencies were calculated at the same level of theory (DFT/B3LYP/6-31+g*). The calculations showed 369 (3 × 125 − 6) internal vibrations (with symmetry of A1g, A2g, Eg, T1g, T2g, A1u, A2u, Eu, T1u) for the 125-atom model of salt. In the O*h* point group, only vibrations with symmetry T1u are active in the infrared spectrum. The frequency and intensity of these 29 IR active T1u vibrations are presented in Table 3. As is shown in Table 3, IR absorption of LiF, LiCl and LiBr appears only in the ranges of 609.41–111.43, 382.61–63.66 and 386.6–42.7 cm^−1^, respectively. Thus, the calculations confirmed that lithium fluoride, chloride and bromide do not absorb infrared radiation in the broad spectral range. The vibrations with the strongest intensity appear at 446.3, 478.2 and 516.3 cm^−1^ for LiF, at 241.0, 299.2 and 332.7 cm^−1^ for LiCl and at 229.1, 303.0 and 371.0 cm^−1^ for LiBr. 

**Figure 2:**
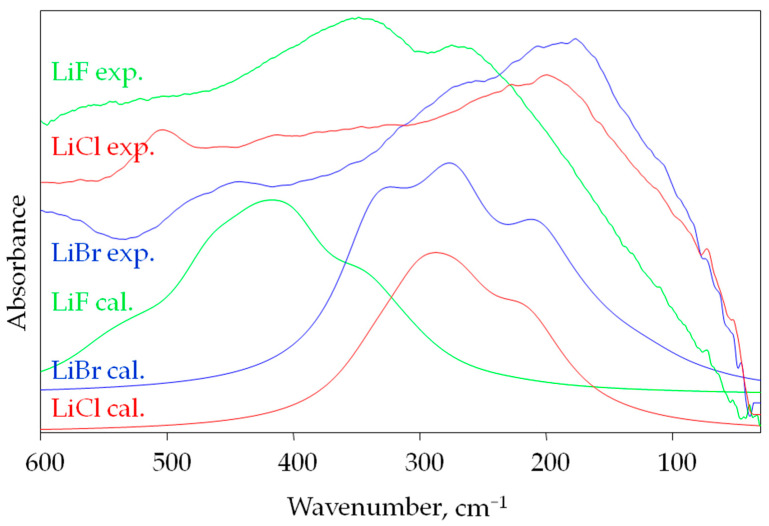
A comparison of experimental and calculated (half-bandwidths 50 cm^−1^, scaling factor = 0.9) infrared spectra for LiF, LiCl and LiBr in the spectral range of 600–30 cm^−1^.

A comparison of the experimental and calculated far-infrared spectra of lithium halides is presented in the range of 600–30 cm^−1^ in Figure 2. 

Figure 2 shows far-infrared spectra of anhydrous LiF, LiCl and LiBr salts. In each case, very broad bands are observed with maximum absorption at 170, 200 and 353 cm^−1^ for LiBr, LiCl and LiF, respectively. Other alkali metal halides also absorb within a similar spectral range. For example, the most intense band in the FIR spectrum of NaCl and KCl is observed at 175 cm^−1^ and at 145 cm^−1^, respectively [41]. The calculation method used has its limitations. In order for the calculated spectra to better reflect the experimental spectra, we used a scaling factor (SF = 0.9) and gave the calculated signals a larger half-width (50 cm^−1^). The experimental spectra (Figure 2) show that the bands are actually wide. The calculations reflect the experimental data well. In particular, they reproduced very well the fact of the high similarity of the LiCl and LiBr spectra and the high contrast of these spectra with the LiF spectrum. 

## 4. Conclusions

The paper presents research on lithium compounds (LiF, LiCl and LiBr), whose importance in the industry is increasing. Although many works devoted to this topic, especially in the last decade, there are still gaps in the current knowledge. The aim of our article was to present additional experimental and theoretical data on the structure and spectral properties of selected anhydrous lithium halides.

The study included an experimental part that involved measurements of far-infrared spectra. To our knowledge, FIR spectra of anhydrous LiF, LiCl and LiBr salts have been presented for the first time. The spectra are unique for each compound; therefore, they can now be distinguished.

The second part of the research consisted of calculations using the density functional theory method. Calculations were performed for a crystal model containing 125 atoms. In each case, the final geometry of the model preserved O*h* symmetries. Theoretical values of lattice parameters and charge distribution in theoretical structures of anhydrous LiF, LiCl and LiBr were calculated at the B3LYP/6-31+g* theory level. The obtained values are consistent with the experimental data presented in the literature. Additionally, in this work, the infrared spectra for the model of crystals of LiF, LiCl and LiBr were calculated (the B3LYP functional and the 6-31+G* basis set) for the first time. The obtained theoretical spectra were similar to the experimental spectra.

Electron charge density distribution and related properties are important to the understanding and design of materials, as many fundamental properties relevant to a wide range of applications are directly related to them. Thus, our research could be useful in predicting the properties of lithium compounds.

## Figures and Tables

**Table 1:** Comparison of experimental and calculated (DFT/B3LYP/6–31+g*) lattice parameters for LiF, LiCl and LiBr.

Chemical Formula	Crystal System	Symmetry Space Group	Unit Cell Parameter	
			*a* = *b* = *c* (Å)	*α* = *β* = *γ* (°)
LiF	isometric	Fm-3m	4.020 ± 0.002 ^a^, 3.906 ± 0.015 ^b^	90.0
LiCl	isometric	Fm-3m	5.143 ± 0.006 ^a^, 5.038 ± 0.022 ^b^	90.0
LiBr	isometric	Fm-3m	5.489 ± 0.006 ^a^, 5.283 ± 0.003 ^b^	90.0

**a**—experimental value [50], **b**—calculated mean value (for 264 distances in model)

**Table 2:** Comparison of calculated ATP charges for LiF, LiCl and LiBr.

Chemical Compound	Li Mean Value (N = 63)	Li Central Atom (N = 1)	Li Surface (N = 13)	X (F,Cl,Br) Mean Value (N = 62)	X (F,Cl,Br) in Direct Surrounding Central Atom (N = 6)	X (F,Cl,Br) Surface (N = 12)
LiF	0.8227 ±0.0176	0.8171	0.8342 ±0.0222	−0.8198 ±0.0057	−0.8169 ±0.0000	−0.8212 ±0.0065
LiCl	0.7831 ±0.0243	0.7626	0.8001 ±0.0289	−0.7796 ±0.0070	−0.7752 ±0.0000	−0.7822 ±0.0076
LiBr	0.7449 ±0.0276	0.7172	0.7645 ±0.0319	−0.7408 ±0.0046	−0.7398 ±0.0000	−0.7412 ±0.0051

**Table 3:** Comparison of calculated (DFT/B3LYP/6-31+g*) IR frequency and intensity of T1u vibrational modes for 125-atom models of halides: LiF, LiCl and LiBr.

No.	LiF		LiCl		LiBr	
	Frequency (cm^−1^)	IR Int. (KM/Mole)	Frequency (cm^−1^)	IR Int. (KM/Mole)	Frequency (cm^−1^)	IR Int. (KM/Mole)
1	111.4	1.7	63.7	2.3	42.7	0.3
2	160.1	0.1	89.6	0.0	67.0	1.6
3	184.2	0.0	101.0	0.0	73.1	1.9
4	213.4	0.9	118.6	0.0	81.3	20.2
5	217.6	0.5	121.0	0.0	84.7	0.0
6	235.3	3.0	130.3	1.9	95.1	0.4
7	250.4	25.8	143.5	15.0	107.1	0.7
8	253.2	0.0	146.1	0.5	110.7	9.4
9	255.4	0.1	158.7	1.5	121.0	38.4
10	265.3	2.1	159.8	7.3	124.7	35.4
11	280.3	0.7	172.3	0.1	137.6	2.8
12	292.1	4.0	174.2	4.6	142.0	111.1
13	314.0	5.5	183.0	1.5	159.1	37.5
14	315.8	8.7	195.0	13.5	173.9	0.0
15	335.1	4.9	201.3	14.5	175.3	119.1
16	341.2	161.5	205.3	0.0	194.5	201.3
17	346.0	142.6	210.7	28.4	215.9	10.9
18	357.4	56.2	212.0	2.3	229.1	1212.2
19	365.0	56.8	214.0	91.0	240.1	3.7
20	377.6	896.1	219.9	4.7	247.5	0.3
21	384.4	106.3	231.5	176.6	266.2	51.1
22	394.8	403.0	236.5	326.1	272.4	9.7
23	439.3	497.7	241.0	1168.7	279.6	14.3
24	446.3	1561.7	277.1	155.2	303.0	1647.0
25	478.2	1570.5772	299.2012	1661.4200	319.8562	40.7289
26	516.3	1549.7978	320.8487	37.8190	332.3225	247.1439
27	529.9	164.5545	332.7207	2017.2156	345.7773	60.3065
28	579.3	476.5014	369.7691	766.1095	371.0345	1523.0904
29	609.4	438.1410	382.6068	75.2121	386.6431	59.1295

## Data Availability

The data presented in this study are available on request from the corresponding author.
